# Influence of brushing with natural dentifrices on color change: In vitro study

**DOI:** 10.4317/jced.58066

**Published:** 2021-08-01

**Authors:** Helena Dutra, Isabel Barbosa, João-Victor Câmara, Gisele Pereira

**Affiliations:** 1 MSc, Department of Dental Clinic, School of Dentistry, Federal University of Rio de Janeiro, Rio de Janeiro, RJ, Brazil; 2 PhD, Department of Restorative Detistry, Piracicaba School of Dentistry, Campinas State University, Piracicaba, São Paulo, Brasil; 3 Master student, Department of Biological Sciences, Bauru School of Dentistry, University of São Paulo, Bauru, São Paulo, Brazil; 4 Adjunct Professor, Department of Dental Clinic, School of Dentistry, Federal University of Rio de Janeiro, Rio de Janeiro, RJ, Brazil

## Abstract

**Background:**

To evaluate *in vitro* the influence of daily brushing with the use of natural toothpastes on the color change of enamel in bovine teeth.

**Material and Methods:**

Four dentifrices were used, one conventional Colgate Total 12 - Clean Mint (G1), and three natural, Contented Toothpaste with Organic and Natural Ingredients (G2); Dental Toothpaste (G3) and Aliv-Gaia Toothpaste (G4). Eighty bovine teeth were distributed in four experimental groups with 20 teeth each (n = 20). The buccal enamel surface of the teeth was subjected to brushing, with the related dentifrices of each group, for 2.13 seconds three times a day, with an electric brush Oral B 5000 Professional Care. Before and after brushing, color measurement tests with a spectrophotometer were performed. The color variation was calculated using the formula ΔE = [(ΔL *) 2+ (Δa *) 2+ (Δb *)2] 1/2. The results obtained were tabulated and submitted to the Kruskal Wallis non-parametric test.

**Results:**

The color change (ΔE) observed was 7.551 and p-value equal to 0.056, determining that there was no statistically significant difference between the groups. However, qualitative tests showed the clearing of all experimental groups, G3 with the greatest change, followed by G2, G1 and G4.

**Conclusions:**

The evaluated dentifrices were not able to promote color change.

** Key words:**Dental enamel, plant extracts, saliva, artificial, dentifrices.

## Introduction

The category of natural oral hygiene products mainly offers ecological toothbrushes, mouthwashes and toothpastes. Natural toothpastes usually contain plant extracts in the role of antibacterial and / or antifungal agents (1). Often these active ingredients have some type of coloring (2). Allied to this, some take in their composition abrasive minerals to help remove stains during brushing (3).

Extrinsic stains tend to form in areas on teeth that are less accessible to daily brushing and, consequently, to the action of abrasives contained in toothpastes (3,4), of which the most used are hydrated silica and calcium carbonate. These have the function of removing the stains, originating from extrinsic factors, through the mechanical movement of brushing (3-5). The side effect related to abrasives is the wear of the tooth structure and the increase in surface roughness, enhanced by an inadequate brushing and the brush type (6,7).

Most natural pastes contain non-industrialized and synthetic substances. Due to the possible staining and abrasion potential of these components, it is extremely important to evaluate the influence of the use of these products in changing the color of the dental enamel. With this in mind, the objective of this study was to evaluate the color change of bovine enamels, through the use of the spectrophotometer, before and after brushing with a conventional and three natural toothpaste. The null hypothesis tested was that the toothpastes used did not influence the color change of the dental enamel.

Materials and Methods

-Materials

This study is experimental *in vitro*. For its realization, four dentifrices were used, one conventional: Colgate Total 12- Clean Mint (Colgate-Palmolive, Industrial LTDA, SP, Brazil) and three natural: Content toothpaste with organic and natural ingredients (organic) Natural, Indústrias Suavetex Ltda, Uberlândia, MG, Brazil); Pródente Toothpaste (Apiário Esperança, MG, Brazil); Aliv-Gaia Toothpaste (Aliv, Vargem Grande, RJ, Brazil) and artificial saliva (Manipulation Pharmacy of the Federal University of Rio de Janeiro, Rio de Janeiro, RJ, Brazil) for the storage of bovine teeth. For brushing, the B Professional Care 5000 oral electric brush was used (Table 1).

**Table 1:**
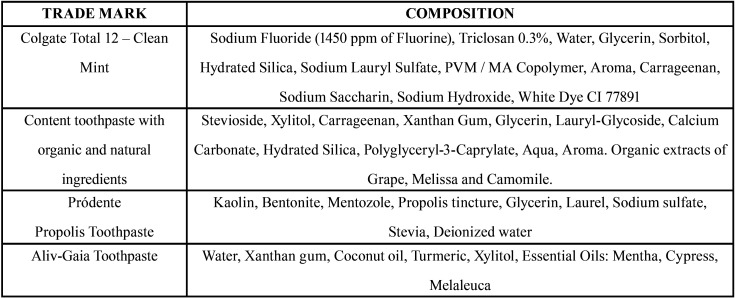
Trademark and components of toothpaste.

-Teeth getting, cleaning and storage

Eighty healthy bovine incisor teeth were obtained (Frigorífico Mondelli, Indústria de Alimentos S / A, Santa Teresa, Bauru, SP.) The teeth were stored for a period of one month in thymol solution 0.1% (UFRJ– CCMN- Department of Biochemistry, Rio de Janeiro- RJ- Brazil) pH 7 at a temperature of 37 ° C for disinfection until the beginning of specimen selection. Of these one hundred and twenty-six bovine teeth, eighty teeth were selected. The exclusion criterion was the presence of cracks and/or defects on the enamel surface.

After the selection of the specimens, the cleaning process of the external surfaces was initiated, by scraping with Gracey periodontal curettes number 7/8 (Hu-Friedy do Brasil - Rio de Janeiro, RJ, Brazil). The objective of this step was to promote the removal of periodontal tissue debris from each dental element. The second stage was prophylaxis with pumice (SSWhite, Rio de Janeiro, RJ, Brazil) and water, using Robinson brushes (KG Sorensen, Barueri, SP, Brazil) mounted at low angle contra-angle (Kavo do Brazil, Joinville, SC, Brazil). After cleaning, the teeth were stored in distilled water until the enamel polishing started.

-Polishing the tooth enamel

To prepare the specimens, the buccal surfaces of the teeth were sanded sequentially with silicon carbide sandpaper (Carborundum/3M do Brasil Ltda., Sumaré, SP, Brazil), with granulations # 400 and # 600, and polished with carbide sandpaper. of silicon # 120023 in a rotating electric polisher (Aropol VV, Arotec, Cotia, SP, Brazil) water-cooled, with a standardized time of 60 seconds for each sandpaper, taking care to regularize the surface throughout its extension.

-Experimental Groups

The teeth remained immersed in distilled and deionized water for a maximum of 24 hours. After this period, they were dried with absorbent paper, identified and randomly distributed (n=20/group): G1 - Conventional - Colgate Total 12 Clean Mint; G2 - Natural Organic; G3 - Pródente, Propolis Toothpaste and G4 - Aliv-Gaia Toothpaste.

Group 1: Twenty ox teeth were brushed with an Oral b Professional Care 5000 electric toothbrush using Colgate Total 12 Clean Mint toothpaste for 45 days, 3 times a day, totaling 6.4 seconds per tooth surface per day.

Group 2: Twenty ox teeth were brushed with an Oral b Professional Care 5000 electric toothbrush using Natural Organic toothpaste for 45 days, 3 times a day, totaling 6.4 seconds per tooth surface per day.

Group 3: Twenty ox teeth were brushed with an Oral b Professional Care 5000 electric toothbrush using Aliv-Gaia Toothpaste for 45 days, 3 times a day, totaling 6.4 seconds per tooth surface per day.

Group 4: Twenty ox teeth were subjected to brushing with an Oral b Professional Care 5000 electric toothbrush using Pródente Toothpaste from Propolis, for 45 days, 3 times a day, totaling 6.4 seconds per tooth surface per day.

After the distribution of dental elements in groups, they remained in 4 plastic organizing boxes with 20 divisions each, one for each tooth, immersed in 25 ml of artificial saliva changed daily, being removed from this solution for the initial and final test. with spectrophotometer; and for daily brushing for 45 days.

-Brush protocol

The brushing of the buccal enamel surface of the dental fragments was performed three times a day by a single operator, with an electric toothbrush,8 soft and rounded bristles, with oscillatory movements of 8,800 rpm, in Daily Cleaning mode, recommended by the manufacturer as daily brushing. The force used in brushing was standardized, since this brush has an alert sensor when there is an increase in pressure.

The brushing of each group was carried out with a suspension containing 33% of the toothpaste diluted in distilled water (9). To standardize the volume of the toothpaste and distilled water, a plastic shell-type meter was used, in the proportion 1:2. The time spent brushing each element was calculated in order to obtain a greater similarity with the recommended period for daily brushing. According to Arnold *et al*. (10), considering 28 teeth per oral cavity = 56 surfaces, simulating the recommended brushing time of 360 s per day (2 minutes each, 3 brushings per day) resulted in a brushing time of 6.4 s per tooth surface. Each group had a portion referring to individualized bristles. At the end of each procedure, the fragments were washed with water / air spray and again stored in 25 ml of artificial saliva for the rest of the time (11-13).

-Color analysis

To measure the color of the dental elements, an objective method (machine) was chosen, the spectrophotometer Vita Easyshade® Advance 4.0 (VITA Zahnfabrik H. RauterGmbH & Co, Bad Säckingen, Germany), instead of a subjective one (eye human), because it presents better precision and gives more data to the analysis (13). For the first part of the study, a mold was made with addition silicone (Variotime - Heraeus Kulzer, Hanau, Germany) on the buccal surface of a single specimen with the purpose of standardizing the positioning during the reading with the spectrophotometer. Color analyzes were performed by a single operator, in order to reduce the influence of natural light, in the same place over artificial light.

These measurements took place on the buccal enamel surface of the teeth in two moments: the first occurred before the beginning of the cupping; and the second after the 45-day period, at the end of brushing.14 For the color readings, the bovine teeth were placed in the addition silicone mold for standardization. A Vita Easyshade® Advance 4.0 spectrophotometer previously calibrated according to the manufacturer’s recommendations was used.

The color was determined using the parameters quantified in the CIE Lab system with three coordinates: L*, (a *) and (b*), where L* describes the tooth value on a scale from 0 (black) to 100 (white) ), (a *) is the measurement along the red-green axis, with (a*) positive indicating red and (a*) negative indicating green; and (b*) is the measurement along the blue-yellow axis, with positive (b *) indicating yellow color and (b*) negative indicating blue color. The color comparison before and after treatment is determined by the difference between the two color shots (ΔE), which was calculated using the formula: ΔE = [(ΔL *) 2+ (Δa*) 2+ (Δb*)2] 1/2. When ΔE is greater than 3.7, an easily visible difference is considered. When it is between 3.7 and 1, it is considered a clinically accepTable difference, and when the ΔE is less than 1, the difference was considered clinically unnoticeable (15).

-Statistical analysis

The values obtained in the color readings, with a spectrophotometer, before and after the brushing period, were tabulated and analyzed statistically by the IBM SPSS 22 software (IBM Corporation, Armonk-NY, United States).

First, the histogram of the data was observed and the Shapiro-Wilks test was performed to assess normality. Due to a strong deviation from normality, the Kruskal-Wallis non-parametric test was used to assess differences between groups. For all analyzes, a significance level of 5% (*p* <0.05) was considered.

## Results

The values obtained in the readings before and after the *in vitro* study, were submitted to statistical analysis. These were made with IBM SPSS 22 software (IBM Corporation, Armonk-NY, United States). For all analyzes, a significance level of 5% (*p* <0.05) was considered.

**Figure 1:**
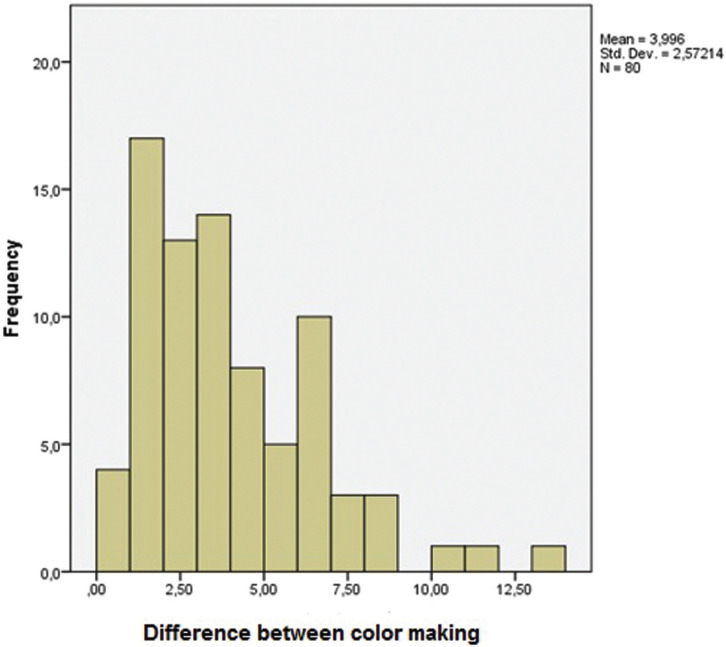
Color change values (ΔE) of the different experimental conditions performed in this *in vitro* study.

The null hypothesis tested initially says that the variable of interest was normally distributed, whereas the alternative hypothesis tested initially, the variable of interest was not normally distributed. First, the histogram of the data was observed and the Shapiro-Wilks test was performed to assess normality (Table 2) (Fig. 1).

**Table 2:**
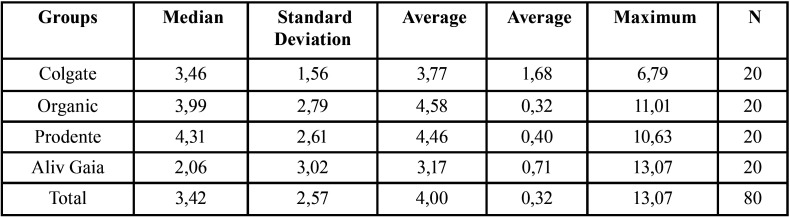
Color change values (ΔE) of the different experimental conditions performed in this in vitro study.

The Shapiro-Wilks test (*p* <0.05) obtained a result of E = 0.92 and *p-value* <0.001, which resulted in a statement with a significance level of 0.5%, that the data were not normally distributed. Thus, the null hypothesis was rejected and the alternative hypothesis, that the variable of interest is not normally distributed, was accepted.

**Figure 2:**
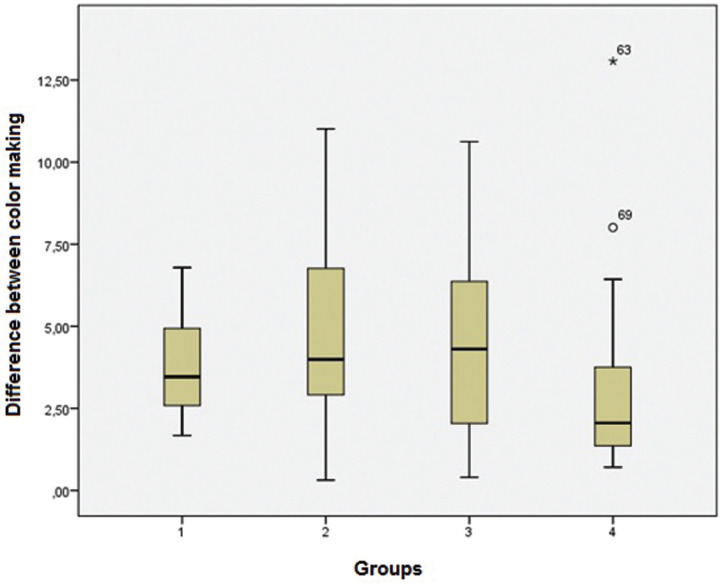
Box-Plot graph showing the median, the interquatile range, minimum and maximum values, and extreme values for the different groups used in the *in vitro* study.

 Due to this strong deviation from normality, the Kruskal-Wallis non-parametric test (Fig. 2) was used to assess the differences between the experimental groups.The null hypothesis in this test was that there was no difference between groups and the alternative, that there was a difference between groups.

The Kruskal-Wallis non-parametric test obtained a result of 7.551 and *p-value* = 0.056. This data did not allow us to reject the null hypothesis, which indicated that there was no statistically significant difference between the experimental groups, thus rejecting the alternative hypothesis.

However, in a qualitative analysis of the difference in color shots, all four experimental groups showed changes between the initial and final color. Table 3 describes the total number of teeth involved in the study, those that clinically had no noticeable change, those with clinically acceptable changes and those that showed easily visible differences.

**Table 3:**
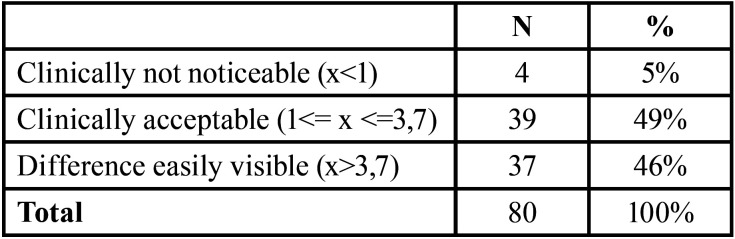
Percentage difference between color shots according to descriptive classification.

With the result obtained in the qualitative test, it was found that 46% of the teeth had a color change classified as easily visible. To confirm the intensity of this difference (in the lightening or darkening of the shade), we used the data recorded in specific worksheets for each of the experimental groups during the study. These data were provided by the color measurement device, the spectrophotometer, which in one of its system parameters, also quantifies the tooth color related to the shade established by the Vita Classical scale. After checking the result of each tooth, whitening of all specimens was observed.

## Discussion

To measure the color of teeth, two types of methods are usually used, one subjective and / or one objective. The subjective is done by the professional with the help of color scales, this compares the color of the natural tooth to be worked with the tabs of the guide, until obtaining an equal and / or similar color. Many variables can compromise the result of this choice, such as the lighting of the office, the reflection of nearby objects, such as a color of clothing, makeup, the angle of vision of the natural tooth, and the main one, the evaluator’s perception in determining the color (13).

In this study, the color evaluation tests were done objectively, using an instrumental method, the spectrophotometer. This makes the procedure faster and more accurate, and can reproduce the previous measurement by 80%, while the human eye is no more than 65% (16). The study by Horn *et al*. (17) in 1998 says that this is more predictable and reliable than the technique subjective assessment of color. In 2009, Judeh *et al*. (18) compared the digital and visual method, and the objective method was five times more likely to be correct when compared to the subjective method.

For a satisfactory evaluation of the data, the spectrophotometer needs to be correctly positioned on the dental structure, so that it occurs with adequate precision (13), making this type of measurement of color data a safer, more accurate and reliable way (15-18). Thus, a matrix made of silicone was used for the standardization of the evaluation site, and the correct positioning on the tooth.

Also, in studies to assess color changes, bovine teeth are often used as an alternative to human teeth in the laboratory, as they have a larger flat area, which can better accommodate the straight end of the spectrophotometer (19,20). Teeth bovines also do not differ significantly from human teeth in terms of the color of the baseline (20).

The aesthetics of teeth receives great importance in Dentistry, being one of the main requests of patients to the professional in the dental office (2). It is known that extrinsic pigmentation is related to the absorption of dyes, from exogenous sources, such as solutions used in eating habits and / or oral hygiene (21,22).

It is important to emphasize that the inherent roughness of the cracks and porosities, contained in the surfaces of the dental elements, can contribute to the increased susceptibility of the tooth to the staining caused by certain solutions (20,22). This occurs, due to the penetration of pigments by the cracks and tubules, present in dental substrates, which can cause an incorporation of these substances into their organic content. It is assumed that agents in liquid form could diffuse more easily through these failures of dental structures (20,22).

The three natural pastes used during the study show some kind of color from the different substances included in their content. The Organic Organic, with a beige color, possibly from the organic extracts of grape, melissa and chamomile contained in its composition.

Pródente has propolis tincture as its main ingredient, with antimicrobial, analgesic, anti-inflammatory, antifungal, and even antitumor action (23). Although it has numerous benefits, it has a dark brown color, which probably pigmented the toothpaste formula. In addition, one of the types of clay that act as abrasives in the paste, bentonite, is also called green clay for its coloring (24).

Finally, Aliv Gaia, brings turmeric as one of its main ingredients. This natural asset has several properties, such as being antioxidant, non-toxic, analgesic, anti-inflammatory, antiseptic and anti-carcinogenic activity (25). Its curcuminoid pigment is gold yellow, which brought the color of the toothpaste. Turmeric, which is a popular food, is often used as the natural food color of choice for replacing artificial colors (26).

Colgate Total 12 toothpaste was chosen as the control group for the study. It is white in color and has only one abrasive in its composition, hydrated silica. These factors lead to the supposition that there could be a possible change in the color of the teeth with the continuous use of these pastes. Since an accumulated exposure to dyes can result in damage to the tooth surface (20). which raises concerns about the effect of these pigments on the color of dental elements (21).

The study was done under specific conditions, the enamel was polished to reduce the grooves and / or defects that could cause some type of alteration in the test, in addition to granting a greater standardization in the specimens used (27). There was no type of pigmentation; previous prophylaxis was performed in order to reduce possible extrinsic stains and the teeth were immersed in artificial saliva to keep their hydration conditions sTable (27).

The results obtained indicated that there was no statistically significant difference between the groups. That is, the four toothpastes used in the *in vitro* study were not able to promote a significant color change in the dental elements.

However, based on qualitative data, there was a color change in all four experimental groups, which should have remained stable. The four groups cleared up. Only 5% of the 80 teeth showed a clinically unnoticeable color difference, that is, they remained stable, while 49% suffered clinically acceptable changes and the other 46%, easily visible differences.

Mechanized toothbrushes with oscillating rotation action are widely used, these show more benefits in terms of reducing gingivitis and plaque when compared to manual toothbrushes (28). However, a 2017 study (7) concluded that electric brushes have a greater potential for abrasion than manual brushes, which may be associated with loss of tooth structure.

The abrasive wear of rigid dental tissues is closely related to the type of brush used, stiffness of the bristles, modulus of force exerted on brushing and the abrasive potential of the toothpaste (6). The present study used an electric brush with a pressure sensor and soft bristle refill. However, due to the qualitative result and the conditions in which the test was submitted, it is assumed that the lightening of the experimental groups may have occurred due to the more abrasive electric brush set contained in the toothpastes under analysis.

The abrasive systems present in dentifrices have the function of ensuring the removal of stains from the surfaces of teeth, due to extrinsic factors. Its undesirable side effect is the wear and tear of dental structures (3,7,29). Abrasives are insoluble substances, such as silica, metal oxides, alumina, phosphates, carbonates and silicates (30). The most used in the industry for the formulation of toothpaste are hydrated silicas and calcium carbonate (28). Studies have shown that what determines a higher value for the degree of abrasiveness are the physical characteristics of the minerals that make up the toothpaste, such as its shape and particle size (4,31). In order of the largest color change to the smallest, the experimental groups were presented as follows: Group 3 - Prodente, Group 2 - Natural Organic, Group 1 - Colgate and Group 4 - Aliv-Gaia. The reasons that lead to suppose the explanation for these results are in the composition of the 4 folders.

Pródente has two types of clay as an abrasive agent in its formula, kaolin and bentonite. These make up a group of very fine clays, formed essentially by particles of crystals (24). Until the present study, no research has been found that relates the use of these clays in brushing. Due to the possible non-standardization of the size and shape of these particles, it can be assumed that they are more abrasive than the components normally found in conventional pastes.

The Organic Organic in turn contains two types of usual abrasives combined, hydrated silica and calcium carbonate. Hydrated silica is a small, regular-shaped particle that is less abrasive than calcium carbonate (4,32). However, the combination of these two substances can increase the abrasive power of these compounds (4,5).

Colgate Total 12 contains only a hydrated silica abrasive, which may have generated lower values when compared to groups 2 and 3. Finally, Aliv-Gaia, a paste used in group 4 specimens, was the one that showed a result smaller compared to other dentifrices. Turmeric as it is found in powder form (26), could cause some type of abrasion. However, like clays in Group 3, no study on the use of turmeric in brushing was found.

Another substance that contributes to the reduction of staining contained in conventional pastes is detergents, such as sodium Laurel sulfate (3). This acts to decrease the surface tension of the composition, increasing the removal of debris, since it can penetrate the grooves on the surface of the enamel (3). In natural pastes this substance may be present or appear modified.

The Natural Organic paste has in its composition the Laruril glycoside a vegeTable detergent, the Pródente although said as natural, contains the Laril sodium sulfate itself, detergent contained in most conventional pastes (3).

Aliv-Gaia is the only paste in the study that initially does not contain any type of detergent, other than natural substances. However, it has in its composition coconut oil, a substance that has lauric acid, which when reacting with the sodium hydroxide contained in saliva, forms sodium lauryl, a type of detergent that can help in reducing plaque (31).

Peedikayil *et al*. in 2015 (31), and Jithender *et al*. in 2017 (33) show that the use of coconut oil showed a significant reduction in plaque indices. It is assumed that this process occurs due to its high saponification value, coconut oil it is one of the most used in the industry for the manufacture of soap. Which justifies the results found for this toothpaste, which obtained a result of whitened teeth in the clinically accepTable classification, but not easily visible because it does not have abrasiveness like the others.

## Conclusions

The dentifrices used did not significantly change the enamel color of the bovine teeth under study. Based on the qualitative test, the abrasion of the dental surface from the abrasive components present in the toothpaste, may have been one of the reasons for the bleaching of the groups, however, to confirm this hypothesis, it would be necessary to carry out micromechanical tests of roughness.
